# Mississippi Valley Association of Dental Surgeons

**Published:** 1844-09

**Authors:** 


*Mississippi Valley Association of Dental Surgeons* —A convention of dentists was recently held in Cincinnati, Ohio, which resulted in the formation of the above named society, and from the professional reputation and standing of many of the gentlemen composing it, the most happy results may be anticipated from the organization. We are personally acquainted with a number of the members of this society, and know them to be men of science and worth. Having thus engaged in the work of reform, and united their energies in the cultivation of the science and art of dentistry, we hope they will go on and co-operate heartily and zealously with the American and Virginia Societies, in their efforts to give character and respectability to the dental profession. The necessity of doing something to elevate its standing and purge it from empiricism, has become so apparent that it would be culpable to remain inactive any longer. Empiricism is laboring assiduously day and night. It has already taken deep root and is spreading its evils abroad over the length and breadth of our land, and to expose and eradicate it from the ranks of the profession, a clear line of demarkation must be drawn between the man of science and the ignoramus, and this can be done in no other way than by concert of action, and by elevating the standard of excellence and by furnishing facilities to those who may hereafter enter the profession, of acquiring a thorough knowledge of it.

We rejoice at all the efforts that are making for the accomplishment of this object, and we extend to our brethren of the Mississippi Valley Association of Dental Surgeons, the right hand of fellowship, and say, God prosper them in their noble and praiseworthy undertaking.

We have been promised the proceedings of this association, but have not as yet received them, and the only account which we have seen of its doings, is the following, which we clip from the Cincinnati Daily Atlas.

Messrs Editors.—It cannot fail to interest the community at large to know, that a convention of dentists was recently held in this city in the Medical College, where addresses were delivered by Drs. M. Rogers, James Taylor, and J. Allen of this city, and a society formed under the name of "The Mississippi Valley Association of Dental Surgeons."

The object of this Association is *to "elevate the standard of Dental Science;"* an object which commends itself at once to all who are interested in the prosperity of the west, and wish to be protected from the impositions of ignorant pretenders. Dr. Rogers' address was characterized by plain, sensible reasoning, the purpose of which was to set forth the importance of raising the standard of dental science, and the necessity of associated action.

The address of Dr. Taylor was admirably adapted to illustrate his subject, "the importance to dentists of a medical education." The traces of classic thought were rich, the practical illustrations forcible, and the whole production evidently the offspring of a well educated mind.

Dr. Allen presented a historical sketch of the dental art from the days of Hippocrates down to the present time. His address evinced deep research into the pages of history, and an intimate acquaintance with the subject.

A large number of respectable dentists from various sections of the country were present, and united in the formation of the society. Several members of the medical profession were also present, and were elected honorary members. Thus, while the arts and sciences generally are improving throughout the west, the dentists, with whom are entrusted in a great degree the health and comfort (not unfrequently the lives) of valuable citizens, have put forth a laudable effort to raise the standard of qualifications for practising their noble art. They have here erected an altar, and laid the foundation of a temple which shall be devoted to science, and which shall stand in future years a proud monument to the memories of those who first publicly stood forward in the cause of humanity and dental surgery.

The following is a list of those who at present constitute the Society:

Dr. J. W. Cook, *President.*
" A. D. Bigelow, 1*st Vice-president.*
" Jos. Taylor, 2*d* *do.*
" D. P. Hunt, 3*d* *do.*
" Wm. B. Ross, *Rec. Secretary.*

Dr. Jas. Taylor, *Cor. Secretary,*

" M. Rogers, " J. Allen, " F. E. Sevier, } *Examining Committee*

" C. Bonsall, *Treasurer.*

*Acting Members.*

Dr. James Clark, Lebanon, O.
" A. Berry, Raymond, Miss.
" J. P. Ulery, Lawrenceburg, Ia.
" P. Knowlton, Cincinnati.
" D. B. Wheeler, do.
" W. R. Winton, Indiana.
" W. H. Goddard, Louisville, Ky.

Dr. C. S. Pleasants, Ohio.
" S. P. Hullihen, Wheeling, Va.
" H. Thomson, Columbus, Ohio.
" B. Strickland, Cleveland, O.
" W. Bashaw, Dayton, O.
" B. B. Brown, St. Louis, Mo.
" W. E. Ide, Zanesville, O.

*Honorary Members.*

L. M. Lawson, M. D. Cincinnati.
M. B. Wright, M. D. do.

W. B. Diver, M. D. Cincinnati.
C. A. Harris, M. D. Baltimore.

In the next Number of the Journal, we hope to be able to give a detailed and correct account of the transactions of the Society.—*Balt. Ed.*

